# Antibody Titer Testing in Dogs: Evaluation of Three Point-of-Care Tests for Canine Core Vaccine Antigens Compared to Virus Neutralization

**DOI:** 10.3390/vetsci12080737

**Published:** 2025-08-06

**Authors:** Lena Janowitz, Ahmed Abd El Wahed, Uwe Truyen, Regina Hofmann-Lehmann, Andrea Monika Spiri

**Affiliations:** 1 Clinical Laboratory, Department of Clinical Diagnostics and Services, and Centre for Clinical Studies, Vetsuisse Faculty, University of Zurich, CH-8057 Zurich, Switzerland; regina.hofmann-lehmann@uzh.ch; 2 Institute of Animal Hygiene and Veterinary Public Health, University of Leipzig, 04103 Leipzig, Germany; ahmed.abd_el_wahed@uni-leipzig.de (A.A.E.W.); truyen@vetmed.uni-leipzig.de (U.T.)

**Keywords:** canine parvovirus type-2 (CPV-2), canine distemper virus (CDV), canine adenovirus (CAV), protective immunity, booster, revaccination, lateral flow test, enzyme-linked immunosorbent dot blot, maternally derived antibodies, duration of immunity

## Abstract

Antibody titer testing can be useful in controlling successful puppy immunization and can reduce unnecessary vaccinations in adult dogs. We evaluated three commercially available point-of-care tests (POCTs) for detecting antibodies against canine parvovirus (CPV-2), canine distemper virus (CDV) and canine adenovirus (CAV-1 and/or -2), comparing them to the reference virus neutralization (VN) assay. Sera from 200 client-owned dogs (13 healthy, 63 chronically diseased, 124 acute) and 60 specific pathogen-free (SPF) dogs, including 20 sera with maternally derived antibodies (MDA), were tested. All three POCTs demonstrated high sensitivity (79.0–100%) and specificity (97.8–100%) for CPV-2. In contrast, specificity for CDV and CAV was lower with POCT-1 (43.5% and 55.3%) and POCT-2 (42.4% and 79.2%), despite high sensitivity (CDV in both POCTs 98.7%; CAV POCT-1: 99.4%, POCT-2: 90.8%). POCT-3, by comparison, showed high specificity (CDV: 94.1%; CAV: 84.4%) but very low sensitivity (CDV: 17.4%; CAV: 33.1%). Only POCT-1 for CPV-2 detected MDA reliably, whereas the other two POCTs, and POCT-1 for CDV and CAV, did not. When compared to VN, the agreement in vaccination recommendations was 82% for POCT-1 and POCT-2, and 62% for POCT-3. In conclusion, all three POCTs reliably detected antibodies against CPV-2, including MDA with POCT-1. However, the lower specificity for CDV and CAV antibody detection in POCT-1 and POCT-2 raises concerns about misclassifying unprotected dogs as immune, while false-negatives with POCT-3 could lead to unnecessary vaccinations. Further optimization of all three POCTs for CDV and CAV is recommended.

## Simple Summary

After initial puppy immunization, guidelines recommend vaccinating dogs every three years against parvovirus, distemper and adenovirus. Studies show that protection often lasts longer. To reduce unnecessary vaccinations, the dog’s protection status can be assessed by antibody testing, offered by specialized laboratories. However, these tests take several days, and specialized personnel and equipment are needed. Rapid tests with quick results for use in the veterinary practice have become available, but have shown reliable results only for the detection of parvovirus antibodies in recent studies. Since then, tests have been revised and a new test has been released. We evaluated the reliability of three rapid tests for detecting antibodies against parvovirus, distemper virus and adenovirus in dogs, compared to the reference method used in specialized laboratories. All tests reliably detected antibodies against parvovirus. However, two of the rapid tests yielded false-positive results for distemper and adenovirus, potentially leading to the misclassification of unprotected dogs as immune. The third test failed to detect antibodies in some dogs, which could result in unnecessary vaccinations. As most vaccine manufacturers provide only combination vaccines covering all three viruses, testing solely for parvovirus offers limited clinical value. Therefore, although there were significant differences among the three tests, each should be further optimized to improve the detection of antibodies against distemper and adenovirus in dogs.

## 1. Introduction

Antibody titer measurements can be a valuable tool in determining appropriate revaccination timepoints and in reducing unnecessary vaccinations. Additionally, dogs with chronic illnesses, a history of adverse vaccine responses, and young dogs can derive benefits from titer analysis [1,2,3]. Identifying young dogs (16 weeks to one year of age) without protective titers—due to the presence of interfering maternally derived antibodies (MDA) at the timepoint of vaccinations or non-responsiveness to vaccinations—is key, as young dogs are more susceptible to disease and experience more severe outcomes than adults [4]. Although prevalences of canine parvovirus type-2 (CPV-2)—an important causative agent of acute enteritis—and canine distemper virus (CDV)—which causes a variety of respiratory, gastrointestinal and neurologic signs—have declined in the last decades in most parts of Europe, cases of parvovirus and distemper infection, even with fatal outcomes, continue to be regularly observed in small animal clinics and practices in Western Europe [5,6,7]. This occurs despite the high prevalence of antibodies among pet dogs in these countries [8,9]. A major contributing factor is the importation of dogs, especially young puppies, from countries with higher infection rates and poor vaccination coverage [7]. In contrast, reported cases of infectious canine hepatitis (ICH), caused by canine adenovirus type-1 (CAV-1, also known as CAdV-1) are very rare. However, if infection occurs, the mortality rate is very high. Outbreaks affecting entire litters have been reported, and the virus can spread rapidly among wild canid species as well [10,11,12]. Current vaccines contain canine adenovirus type-2 (CAV-2, also known as CAdV-2), which provides cross-protection against CAV-1 and thus help prevent ICH [13]. These vaccines also protect against CAV-2 itself, which is primarily associated with respiratory disease and is part of the canine infectious respiratory disease complex (CIRD) with high morbidity but low mortality. CIRD is of particular clinical importance in young and subadult dogs [14]. However, maintaining herd immunity in the adult population can also reduce infection pressure in younger dogs. Thus, all dogs should maintain protective immunity throughout their lifetime against CPV-2, CDV and CAV-1, as recommended by internationally recognized vaccination guidelines [15,16,17]. The appropriate timepoint and frequency of revaccination depend on various factors such as age, lifestyle and environment of each dog [18,19,20]. Studies have also shown that protective immunity can last for many years when modified live virus vaccines (MLV) are administered at the appropriate age [4,21,22].

Assays for antibody detection, such as virus neutralization and hemagglutination assays (the gold standard tests for CDV, CAV and CPV-2) and immunofluorescence tests, are well established, but time-consuming and laboratories with high-standard equipment and personnel are required. Although point-of-care tests (POCTs) provide only qualitative or semiquantitative results and detect binding rather than exclusively neutralizing (confirmed protective) antibodies, POCTs can facilitate titer testing through their accessibility, their speed, and ease of use by on-site personnel at the point of care. Studies have shown that these POCTs reliably detect antibodies against CPV-2 [23,24]. For CDV and CAV, divergent study findings exist: In healthy dogs, one study reported low performance of one POCT [25], while another supported the reliability of the same test [26]. Studies involving primarily sick dogs found that the tested POCTs did not reliably detect antibodies against CDV and CAV [27,28]. And yet another study with a study population of unknown health status indicated reliability of one of the tests [24].

Given the need for optimization of POCTs for titer testing in dogs on the market and the availability of a new commercial test, we aimed to evaluate three different POCTs for their reliability in detecting antibodies, particularly against CDV and CAV in dog sera from healthy, chronically diseased and acutely diseased dogs. The performance of the POCTs was compared to virus neutralization (VN) and for CDV to an additional immunofluorescence assay (IFA).

## 2. Materials and Methods

### 2.1. Serum Samples

Serum samples from 200 client-owned dogs were obtained from the biobank of the Clinical Laboratory of the Vetsuisse Faculty in Zurich. The biobank stores leftover samples from the routine diagnostics of the Clinical Laboratory. The use of sample material in scientific projects has been approved by the Ethics Committee of the Faculty of Medicine, University of Zurich (MeF-Ethik-2024-14). Animal owners gave their consent for the use of data and residual sample material in research prior to the onset of the study. Serum has been collected between April 2023 and June 2024 from 87 female (43.5%) and 113 male dogs (56.5%). Of these, 41 dogs were mixed breed and 159 were purebred, representing 76 different breeds, with Golden and Labrador Retrievers being most frequent (25/159) (Table 1). The age ranged from two and a half months to 17 years and two months, with a median of 7.7 years. Based on medical records and C-reactive protein (CRP) blood levels (reference range: <6.4 mg/dL), dogs were classified into two subgroups: one consisting of healthy dogs (*n* = 13) and dogs with chronic disease but considered eligible for vaccination (*n* = 63), and the other comprising dogs with acute disease (*n* = 124). These dogs were suffering from various medical conditions. CRP is a major acute-phase protein, making it a sensitive marker for detecting acute or ongoing inflammation [29], and was measured by a Cobas 6000 analyzer (Roche, Basel, Switzerland) via immunoturbidimetric assay (Gentian, Moss, Norway).

Furthermore, 40 serum samples from specific pathogen-free (SPF) and specific antibody-free unvaccinated beagle dogs (Isoquimen, S.L., Barcelona, Spain; Boehringer Ingelheim Animal Health, Saint-Vulbas, France) as well as 20 serum samples from SPF dogs with MDA (Marshall Beagle^®^, Marshall BioResources, Lyon, France) were also included. Until testing, all serum samples were stored at −80 °C.

### 2.2. Point-of-Care Tests (POCTs)

Three commercially available antibody POCTs (POCT-1 to POCT-3) were assessed regarding their performance and practicability for point-of-care use: Canine VacciCheck^®^ (Biogal Galed Labs, Kibbutz Galed, Israel), VCheck CPV/CDV/CAV Ab (Bionote Inc., Hwaseong-si, Republic of Korea) and Fassisi CantiCheck Plus (Fassisi^®^, Gesellschaft für Veterinärdiagnostik und Umweltanalysen mbH, Göttingen, Germany) (Table 2). All tests detect antibodies against CPV-2, CDV and either CAV-1 (POCT-1) or CAV-2 (POCT-3) or both CAV types (POCT-2) according to the manufacturers. Since cross-protection between CAV-2 and CAV-1 is evident [13], and a study reported minimal differences in prevalence [28], we chose to measure only CAV-1 in the VN assay (as described below).

POCT-1 measures serum IgG semiquantitatively via an enzyme-linked immunosorbent dot blot assay: Antibodies bind to the specific viral antigen immobilized on a plastic card, referred to as a comb. After washing, an enzyme-labeled anti-dog IgG antibody is added. After a second washing step, a chromophore substrate is added. A positive reaction produces a color change of the dot and different levels of grey intensity can be observed, which are compared to the CombScale (Biogal Galed Labs) from S0 to S6. Each score is approximately equivalent to a titer from the reference gold standard test. Titers equal to or greater than S2 (corresponding to reference test titers of 1:40 for CPV-2, 1:16 for CDV, and 1:8 for CAV) are considered positive according to the manufacturer’s guidelines. POCT-1 is approved by regulatory authorities, including the United States Department of Agriculture (USDA, Washington, DC, USA) and the Canadian Food Inspection Agency (CFIA, Ottawa, ON, Canada).

POCT-2 and POCT-3 are classic lateral flow tests, which consist of a cassette with a test and a control line. While the readout of the POCT-3 is quantitative by eye, the POCT-2 needs a fluorescence detection device (VCheck V200 analyzer, BioNote Inc., Hwaseong-si, Republic of Korea) to detect antibodies labelled with the fluorophore Europium and provides a semiquantitative titer approximately equivalent to the gold standard reference test. For POCT-2, a titer of ≥1:80 for CPV-2, ≥1:32 for CDV and ≥1:16 for CAV is considered positive with a sufficient immune status, where no booster vaccination is recommended.

Each POCT was performed batchwise on all sera and interpreted independently according to the manufacturers’ instructions without knowledge of the other POCT results and before conducting the virus neutralization.

The incubation time for the tests was 10 min for POCT-2 and POCT-3 and 21 min for POCT-1. Thus, total conduction time was 45 min for POCT-2 and POCT-3 (including 30 min for clotting and 5 min for centrifugation to prepare serum samples, and 10 min for incubation), and 81 min for POCT-1, including 60 min of acclimatization to reach room temperature prior to testing. POCT-3 can also be performed using whole blood, reducing the total testing time to 10 min.

The interpretation of POCT-1 and POCT-3 test results is subjective, as it relies on visual assessment. In contrast, the analyzer of POCT-2 automatically displays the results, providing an objective assessment. For POCT-1, objective result interpretation is also possible using the CombCam (Biogal Galed Labs, Kibbutz Galed, Israel), a small device developed to read one tooth of the comb at a time. However, the camera is not validated for the use with POCT-1 and was therefore not used in this study.

The extent of hemolysis, lipemia and hyperbilirubinemia in the serum samples were evaluated with a Cobas 6000 analyzer (Roche, Basel, Switzerland) to assess the quality of sera and identify possible reasons contributing to test invalidities. Manufacturer specifications vary from precise cut-off values to advising against testing in general in case of visible lipemia, hemolysis or hyperbilirubinemia (Appendix A Table A1). However, in private practice, there is often no opportunity to specifically assess serum quality.

### 2.3. Virus Neutralization (VN) Test

All tests were run in duplicates and interpreted under the principle of dual control by experienced laboratory personnel and L.J. The following cells and virus strains were used, respectively: Crandell’s Rees feline kidney cells for CPV-2 (strain vBI 256), Madin-Darby canine kidney cells for CAV-1, and Vero SLAM cells for CDV (strain 219 Ag). Cells were cultured in Dulbecco’s Modified Eagle’s Medium (Biowest, Nuaillé, France) supplemented with 1% nonessential amino acids (Thermo Fisher Scientific, Waltham, MA, USA), 1% Penicillin-Streptomycin (Thermo Fisher Scientific) and 10% fetal calf serum (FCS, Thermo Fisher Scientific) at 37 °C with 5% CO_2_. For seeding into 96-well microtiter plates, cells were washed with phosphate-buffered saline (PBS, pH 7.2) and detached from cell culture flasks using 0.25% Trypsin-EDTA (Thermo Fisher Scientific). Seeding was performed the afternoon prior to the VN test to let cells reattach and recover.

Aliquots of each serum were heat-inactivated (56 °C, 30 min), prediluted 1:10, and subsequently serially diluted 1:2 in 96-well microtiter plates (Thermo Fisher Scientific) up to a final dilution of 1:10,240. Sixty microliters of virus per well, at a concentration of 2 × 10^3^ TCID_50_ per mL, were added to the 60 μL of diluted serum. After incubation (37 °C, 90 min, 5% CO_2_) the serum-virus-mixture was transferred onto 96-well microtiter plates containing the corresponding cells. Cell densities were 1 × 10^4^–1 × 10^5^ cells/mL for CRFK cells, 1 × 10^3^ cells/mL for MDCK+ cells, and 2 × 10^4^ cells/mL for Vero SLAM cells, respectively.

The microtiter plates for CAV and CDV neutralization were observed daily for a cytopathic effect for up to five days with an inverted microscope (Helmut Hund GmbH, Wetzlar, Germany) at 10 × magnification. A cytopathic effect indicates the lack of neutralizing antibodies. Sera containing neutralizing antibodies neutralize the virus, and the cells remain intact. The reciprocal value of the highest dilution, at which no cytopathic effect was observed, corresponds to the antibody titer. Serum from a vaccinated client-owned dog with a known titer served as the positive control, and a SPF dog serum provided by the Institute of Animal Health and Hygiene in Leipzig was used as the negative control. A titer ≥ 10 was regarded positive.

For CPV-2, a distinct cytopathic effect was often invisible with the microscope; therefore, an indirect immunofluorescence test was added to read the test results. After three days of incubation of the serum dilutions with CPV-2, CRFK cells were fixated with ice-cold acetone/methanol (1:1) at −20 °C, dried for 30 min, washed with PBS, blocked with a 3% FCS/PBS solution, and incubated with an anti-CPV2-monoclonal mouse antibody [30] overnight at room temperature. After a single washing step with PBS, a second fluorescein isothiocyanate (FITC)-labelled goat–anti-mouse IgG conjugate (Dianova, Hamburg, Germany) was added. After incubation (room temperature, overnight), the microtiter plates were examined under a fluorescence microscope (Leica DMIL, Wetzlar, Germany). Fluorescence was observed where no neutralizing antibodies were present in the diluted sera, and therefore non-neutralized virus caused a cytopathic effect on the cells. Virus neutralization was conducted at the Institute of Animal Hygiene and Veterinary Public Health of the Faculty of Veterinary Medicine in Leipzig, Germany.

### 2.4. Immunofluorescence Assay (IFA)

Since VN detects neutralizing antibodies, whereas POCTs detect all binding antibodies (neutralizing and non-neutralizing antibodies), an IFA for the detection of antibodies against CDV was also performed. Sera were tested as follows: Vero cells (susceptible to CDV infection) were cultured in RPMI-1640 medium (Gibco/Thermo Fisher Scientific, Waltham, MA, USA) supplemented with 10% FCS (Thermo Fisher Scientific) and 1% antibiotics/antimycotics (100×, Thermo Fisher Scientific) at 37 °C and 5% CO_2_ until they reached 80–90% confluence. After infection with CDV (American Tissue Culture Collection (ATCC) VR-128), cells were detached with 0.25% Trypsin (Thermo Fisher Scientific) and washed with Hanks’ Balanced Salt Solution (HBSS, Thermo Fisher Scientific) as soon as a cytopathic effect was observed. The absence of CAV-1, CAV-2, CPV-2, canine enteric coronavirus, canine herpesvirus, canine parainfluenza virus, canine influenzavirus, canine respiratory coronavirus, tick-borne encephalitis virus, *Anaplasma platys* and *Anaplasma phagocytophilum* of each CDV-infected cell suspension was tested by qPCR/RT-qPCR. An aliquot of the cell suspension containing approximately 1.5 × 10^6^ cells/mL, in a ratio of 80% infected to 20% non-infected cells, was added to each well on Teflon-coated slides (Milian^®^ AG, Bremgarten, Switzerland). The slides were dried under the laminar flow, fixed with ice-cold acetone at −20 °C for ten minutes and stored at −20 °C until use.

Positive and negative controls and samples were diluted 1:80 in PBS. Then, 20 μL of each control and diluted samples were applied to a well on the slides and incubated for one hour at 37 °C in a humid chamber to allow for antibody binding. After three thorough washes with PBS and air-drying, 20 μL of FITC conjugate (rabbit anti dog IgG (H+L)/FITC, Nordic-MUbio, Susteren, The Netherlands), diluted 1:40, was added. Another incubation period of one hour at 37 °C in a humid chamber followed, along with a final washing step of three washes. Slides were covered with a coverslip and examined under a fluorescence microscope with a 450–495 nm wavelength blue light at 400× magnification (Leica DMLB). SPF serum served as a negative control. As a positive control, serum from a client-owned dog with known titers for CDV was used.

Results were considered positive when fluorescing inclusion bodies were observed at or above a 1:80 dilution. Sera were then further diluted twofold to the last dilution that still tested positive to determine the exact titers.

### 2.5. Statistical Analysis

Data were analyzed using GraphPad Prism 10.2.3 for Windows (GraphPad Software, Boston, MA, USA), Microsoft Excel 2504 (Microsoft, Redmond, WA, USA) and MedCalc 23.2.1 (MedCalc Software, Ostend, Belgium). Sensitivity, specificity, positive predictive value (PPV), negative predictive value (NPV) and overall accuracy (OA) were calculated with a 95% confidence interval (CI). Cohen’s Kappa coefficient (κ) was used to determine the level of agreement between the three POCTs. Values for the κ coefficient were interpreted according to Landis and Koch: 0.01–0.20 none to slight, 0.21–0.40 fair, 0.41–0.60 moderate, 0.61–0.80 substantial and 0.81–1.00 almost perfect agreement [31]. Statistical significance was considered at a *p*-value of <0.05.

## 3. Results

### 3.1. Quality of Serum Samples and Practicability of POCTs

Except for two samples (sample IDs 76 and 196), the cut-off values for lipemia, hyperbilirubinemia or hemolysis as specified by the manufacturer of POCT-2 (Table A1) were not exceeded (median/25th and 75th percentile in mg/dL, respectively: lipemia 20/7–73, hemolysis 13/7.3–22, hyperbilirubinemia 0/0–0). The aforementioned samples were both icteric (5 and 6 mg/dL, cut-off value 5 mg/dL), but the POCT-2 analyzer did not display an error code and test results were considered valid.

**Figure 1:**
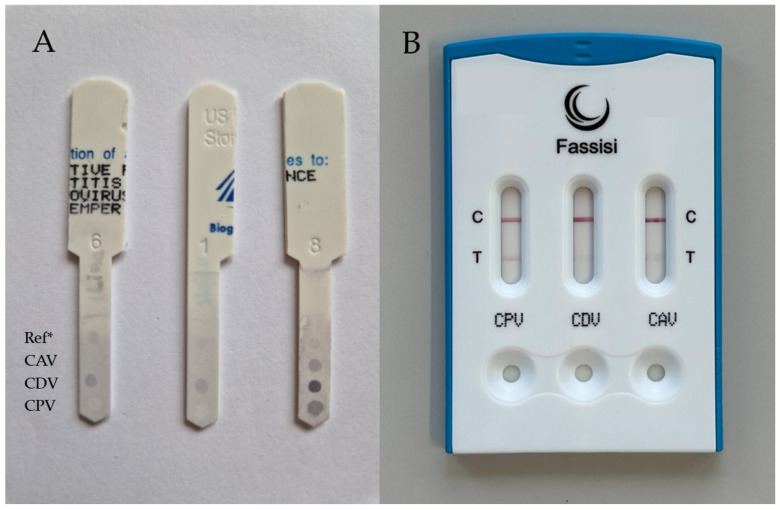
Examples of point-of-care test (POCT) results with difficult or ambiguous results. (**A**) POCT-1 with read-out difficulties; left and middle: a dark background with indistinct reference and test spots is visible in POCT-1; test results are deemed invalid (sample ID 29); right: a dark background with distinct reference and test spots is visible in POCT-1 and the test is considered as valid (sample ID 113). (**B**) Ambiguous test results in POCT-3: POCT-3 showed faint test lines for CDV and CAV, which were considered negative according to the manufacturer’s instructions. CPV was considered positive; note that the control line (C) cannot be used as a reference to interpret test results (T, test line). Ref* (**A**), reference spot; CAV, canine adenovirus; CDV, canine distemper virus; CPV, canine parvovirus.

Therefore, all samples were subsequently analyzed using all three POCTs. POCT-1 exhibited a dark background with indistinct test spots for one sample (ID 29) twice and the test result was deemed invalid (Figure 1A). In 44 cases for CPV-2, in 74 cases for CDV and in 98 cases for CAV, POCT-3 showed very faint test lines, which were interpreted as negative (according to the manufacturer’s instructions) (Figure 1B).

### 3.2. VN Results

**Figure 2:**
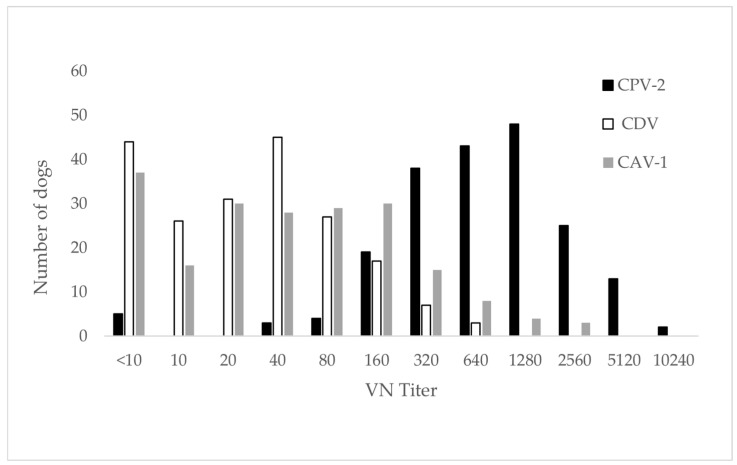
Distribution of neutralizing antibody titers of all tested sera for the three viruses. Antibody titers are expressed as the reciprocal of the highest serum dilution without cytopathic effect; titers < 10 are considered negative.

Raw data related to the performance of all tests can be found in the Appendix A (Table A2, Table A3, Table A4 and Table A5). Neutralizing antibody prevalence, as determined by VN, was 97.5% (195/200) for CPV-2, 77.5% (155/200) for CDV and 81.5% (163/200) for CAV-1 across all client-owned dogs (*n* = 200). For CDV and CAV, the majority of dogs showed low to moderately high titers ranging from 1:10 to 1:640 for CDV and 1:10 to 1:2560 for CAV-1 (Figure 2, Table A2). For CPV-2, titer up to 1:10,240 were detected and most dogs revealed moderately high to very high titers (Figure 2, Table A2).

### 3.3. Performance of POCTs

For CPV-2, specificity was 100% for POCT-1 and POCT-3, and 97.8% for POCT-2, alongside high sensitivity (POCT-1 100%, POCT-2 93.9%, POCT-3 79.0%), with only one false-positive result for POCT-2 (Table 3). Among client-owned dogs, specificity was 80% for POCT-2, whereas POCT-1 and POCT-3 maintained 100% specificity, again with high sensitivity (79–100%). In SPF dogs, all POCTs achieved 100% specificity.

In contrast, specificity for CDV was low for POCT-1 (43.5%) and POCT-2 (42.4%) or high, together with a very low sensitivity for POCT-3 (specificity 94.1%, sensitivity 17.4%) (Table 4). POCT-1 and POCT-2 classified 48 and 49 dogs, respectively, as antibody positive, where virus neutralization did not detect neutralizing antibodies. POCT-3 had five false-positive results. Regarding client-owned dogs only, specificity was even lower for POCT-1 (13.3%) and POCT-2 (17.8%), regardless of each dog’s health status. When considering SPF samples only, specificity was markedly higher (70.0–95.0%).

For antibodies against CAV, specificity ranged from 55.3–84.4%, alongside high sensitivity for POCT-1 (99.4%) and POCT-2 (90.8%) and low sensitivity for POCT-3 (33.1%) (Table 5). POCT-1 revealed the most false-positive results (*n* = 34). In client-owned dogs only, specificity was lowest in POCT-1 (8.3%), followed by POCT-2 (56.8%), both showing high sensitivity. POCT-3 had the highest specificity (81.1%), but a low sensitivity (33.1%). When considering only SPF sera, specificity was markedly higher and ranged from 87.5–100%.

**Figure 3:**
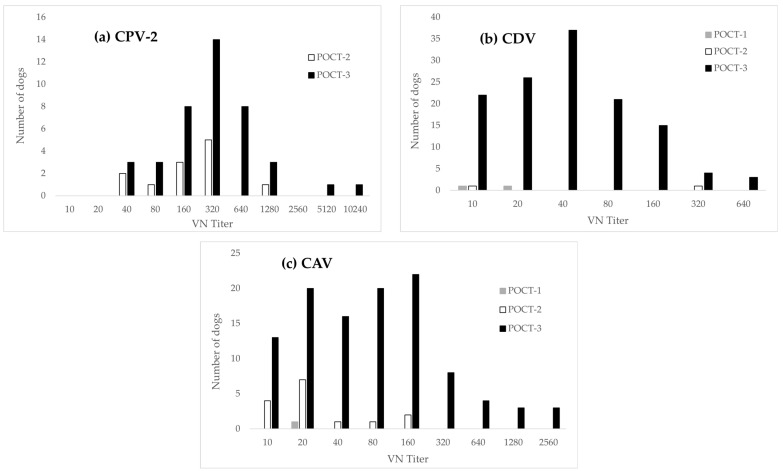
Histogram illustrating the distribution of samples testing false-negative by POCTs, grouped by VN titer levels for (**a**) CPV-2 (**b**) CDV (**c**) CAV. No false-negative results were observed with POCT-1 for CPV-2; therefore, it is not shown in panel (**a**).

Regarding false-negative results, discrepancies between the POCTs and VN were minor for POCT-1 (high sensitivity), but greater for POCT-2 and POCT-3 (Figure 3). For CPV-2 in particular, high VN titers contrasted with negative results from POCT-3 (Figure 3a).

**Figure 4:**
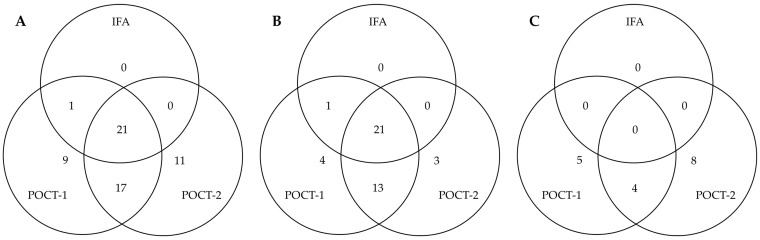
Venn diagram showing the number and distribution of false-positive results (negative in VN) for CDV obtained by POCT-1 and POCT-2 and compared to immunofluorescence assay (IFA). (**A**) All dog sera (without MDA): of 48 false positives in POCT-1 and 49 false positives in POCT-2, 21 sera were also positive in IFA. (**B**) Client-owned dogs only: of 39 false positives in POCT-1 and 37 false positives in POCT-2, 21 sera were also positive in IFA. (**C**) SPF sera: of 9 false positive results in POCT-1 and 12 false positive results in POCT-2, 0 sera were also positive in IFA. POCT-3 showed only five false-positive results, of which one overlapped with IFA, three with POCT-1, and four with POCT-2. Due to the small number of false positives, POCT-1 is not included in the Venn diagram.

False-positive results (sera negative in VN) of POCT-1 and POCT-2 for CDV were further evaluated using IFA, as both POCTs and IFA detect all binding antibodies. A comparison between IFA, POCT-1 and POCT-2 revealed agreement: among 85 sera that tested negative for CDV antibodies by VN, 22 were antibody-positive in the IFA, 48 in POCT-1 and 49 in POCT-2 (Figure 4). In client-owned dogs specifically, the IFA results aligned well with those of the POCTs: agreement was found in 21 cases for POCT-1 and in 22 for POCT-2. In contrast, among SPF sera, IFA detected no antibodies, whereas both POCTs returned positive results. POCT-3 showed only five false-positive results. Of these, one overlapped with IFA, three with POCT-1 and four with POCT-2. Due to the small number of false positives, it was excluded from Figure 4.

Of the total 60 SPF samples, 20 tested positive for antibodies in virus neutralization (Table 6). These 20 samples originated from three-week-old beagle puppies (Marshall Beagle^®^), whose queens received yearly vaccination against CPV-2, CDV and CAV; thus, the antibodies were assumed to be maternally derived. Except for POCT-1 and CPV-2, the POCTs did not show a good sensitivity and specificity for MDAs when compared to virus neutralization (Table 6). For CDV and CAV, POCT-1 exhibited good sensitivity but low specificity. POCT-3 did not detect any MDA with a sensitivity of 0% for all viruses. POCT-2 also revealed many false-negative and false-positive results, with the best performance observed for MDA against CAV.

The level of agreement between the three POCTs regarding test results of all client-owned and antibody-free SPF dogs was dependent on the virus: moderate to substantial agreement was observed between all three POCTs for CPV-2 and also between POCT-1 and POCT-2 for CDV and CAV (Table 7).

To translate the results into practical clinical decision-making, vaccination recommendations were calculated based on test results for all dogs eligible for vaccination. For POCT-1 dogs younger than 20 weeks of age were excluded as the manufacturer recommends using the test only in dogs older than 20 weeks of age. In contrast, for POCT-2 and POCT-3, all 154 cases were included in this calculation (Table 8; POCT-1 *n* = 101, POCT-2 and POCT-3 *n* = 154). Vaccination was advised if at least one antibody titer was considered insufficient. Excluding dogs under 20 weeks of age, 51 of 101 dogs required vaccination (50.5%) according to VN, while 50 dogs (49.5%) showed adequate protection against all three viruses. POCT-1 recommended vaccination for 35 dogs (35%) (Table 8). Including all 154 dogs, 85 (55%) required vaccination, while 69 (45%) showed adequate protection against all three viral diseases according to VN results. POCT-2 recommended vaccination for 77/154 dogs (50%), whereas POCT-3 suggested vaccination for nearly every dog 142/154 (92%) (Table 8). Overall and considering the vaccination decision for each dog individually, recommendations based on POCT-1, POCT-2 and POCT-3 agreed with VN in 82%, 82% and 62% of cases, respectively.

## 4. Discussion

This study was conducted to provide an update on the performance of POCTs in antibody titer measurements for dogs and to independently evaluate a relatively new POCT on the market. We observed remarkable differences in performance between the POCTs depending on the targeted antibodies. The evaluation also considered aspects such as ease of handling, interpretation of results, and the capability to detect maternally derived antibodies. Two POCTs showed low specificity for CDV and CAV, while the third POCT showed high specificity but low sensitivity. For CPV-2 all POCTs showed high sensitivity and specificity.

Since protective titers against CPV-2, CDV and CAV are reported to often last longer than three years, after which revaccination is recommended, antibody titer measurements via POCTs in the veterinary practice would be desirable for reducing unnecessary vaccinations and obtaining quick results, provided the tests are reliable.

The antibody prevalences observed in this study were excellent for CPV-2, acceptable for CDV and CAV, and are associated with adequate herd immunity, assuming a herd immunity threshold of 75% [32,33].

The performance of the POCTs for antibody titer measurements varied between the three evaluated viruses: for the detection of antibodies against CPV-2, all three POCTs, including the newly introduced test (POCT-2), demonstrated an excellent specificity when compared to virus neutralization. These findings are consistent with and extend results from previous studies investigating POCT-1 and POCT-3 [23,24].

In contrast, for the detection of antibodies against CDV and CAV no POCT achieved a high specificity alongside good sensitivity. High specificity is crucial since falsely positive tested dogs would not receive a vaccination, leaving these dogs vulnerable to potentially fatal diseases. With a specificity for CDV of 43.5% and 42.4%, respectively, POCT-1 and POCT-2 misclassified more than every second dog lacking antibodies as protected. In contrast, POCT-3 exhibited high specificity but low sensitivity for CDV and CAV. Certainly, specificity is the most important parameter, but low sensitivity may result in many unnecessary vaccinations. The recommendations for vaccination underline these findings. Vaccination was advised if at least one antibody titer was considered insufficient. In VN, 69% of the dogs exhibited sufficient titers against all three viruses and 31% received a vaccination recommendation. Consequently, the number of vaccination recommendations was higher than anticipated based on the antibody prevalences (78.0–97.5%) for the individual viruses. When compared to VN, POCT-3 tended to underestimate the number of dogs with sufficient immunity, showing an agreement of 62% with VN, whereas POCT-1 and POCT-2 appeared to slightly overestimate the number of dogs with adequate protection, each showing an agreement of 82% with VN.

Sensitivity and specificity depend on the chosen cut-off value, which determines whether results are interpreted as positive or negative. For POCT-1, for example, the manufacturers adjusted the cutoff from S3/positive to S2/weak positive after the release of a new study in 2021 [24]. When interpreting results, which were equal to or higher than S2 as protective and thus positive, specificity for CDV for SPF sera was 79% (the applied cut-off in this study, Table 4). When regarding S2 as not protective and thus negative, specificity would increase to 100% for SPF sera. The same can be applied for the client-owned dogs only, where specificity would increase from 43.5% to 60.0% while the sensitivity decreases from 98.7% to 92.9%. Nevertheless, the increase in vaccination recommendations would be minimal (from 35/101 to 38/101 dogs; 84% agreement with VN), while specificity would still be considered suboptimal. On the other hand, for POCT-3, faint test lines were considered negative according to the manufacturers’ guidelines in this study), resulting in a specificity of 93.3% and a sensitivity of 17.4% for CDV in client-owned dogs (Table 4). When interpreting these faint test lines as positive, the sensitivity would increase to 52.9% but the specificity would decrease to 71.8%.

Another potential reason for the low specificity and sensitivity in general could be the presence of interfering endogenous antibodies, such as heterophile antibodies and canine anti-mouse antibodies, in canine blood [34,35]. These antibodies may have caused unspecific binding with the antigens used in the POCTs, leading to false-positive or—by steric hindrance—to false-negative results. Heterophile antibodies are a common cause for interference in immunoassays, particularly in Sandwich-ELISAs [36,37]. The prevalence of heterophile antibodies in canine blood was reported to be 9% by one study [34]. Other authors suggest it may be as high as 40%, similar to the prevalence observed in humans [38]. Techniques for removing interfering antibodies have been developed [38,39,40] and could potentially enhance the accuracy of POCTs. However, these techniques require highly specialized equipment and cannot currently be performed at the point of care.

The hook effect is another potential cause of false-negative or faint-positive results [40]: It occurs when the concentration of the analyte (in our case CPV-2/CDV/CAV antibodies) is excessively high and surpasses the binding capacity of the labeled detection antibodies. In such cases, excess analyte saturates both the antigen and the detection antibodies independently, reducing signal intensity. In one case of CPV-2, three cases of CDV and three cases of CAV, faint or negative test lines were observed with POCT-3, while VN showed titers at the highest dilution tested (Figure 3, Appendix A Table A3, Table A4 and Table A5). Thus, this effect (saturation) might have contributed to false-negative results in POCT-3. Furthermore, some false negative results in POCT-3 had a low VN titer of 1:10 (22/128 CDV, 13/109 CAV-1), but the majority had titers ≥ 1:20 (Figure 3). Therefore, a high limit of detection (low analytical sensitivity) cannot entirely explain false-negative results, and technical factors such as suboptimal target antigens and/or low-affinity capture antibodies or subpar particle size and concentration of the detection labels might have also contributed to a low (diagnostic) sensitivity [41,42].

Hemolysis, lipemia and hyperbilirubinemia are well-known causes of interference, especially for chemical parameters, but they are less likely to affect antibody titer measurements [35,43]. We did not detect any interference from hemolysis, lipemia or hyperbilirubinemia; thus, we do not anticipate that these factors had an impact on our results. In one case with POCT-1, the serum produced a dark background with indistinct test spots, making result interpretation impossible. According to the manufacturer, hemolysis and, in very rare cases, hyperglobulinemia may cause this dark background; however, neither of these factors was present in this sample.

The majority of our privately-owned study population consisted of acutely ill dogs and dogs with chronic disease (94%). Whether the health status influences the POCTs is unknown, as previously stated [27]. Bergmann et al. also reported low specificity for CDV, ranging from 8–59%, across four different POCTs including POCT-1, as well as for CAV-1 (56% in POCT-1), in a cohort of client-owned, predominantly sick dogs [27]. We assume that heterophile antibodies might be more present in ill dogs. Whether the type of illness (e.g., endocrine, orthopedic, neoplastic) had an impact cannot be answered by this study, but it might explain why the observed specificity for chronically ill dogs was even lower than the specificity for acutely ill dogs (Table 4 and Table 5). In contrast, the observed specificity and sensitivity of all three tested POCTs for detecting CPV-2 antibodies in clinically ill dogs were excellent. Achieving similarly high performance for CDV antibodies would be desirable, as dogs with chronic diseases, in particular, would benefit from titer testing. The observed lower performance for CDV might be attributed to the significant antigenic variability, particularly in the CDV H protein, which plays a key role in antigen recognition and host cell interaction [44,45]. This variability poses a considerable challenge both in vaccine development and diagnostics [46]. In contrast, CPV-2 has undergone minimal changes in its capsid protein over the past few decades [47]. Moreover, CAV also is a rather conserved virus with little antigenetic variability [14].

For POCT-1, divergent study results especially for CDV already exist: One study involving healthy, client-owned dogs due for triennial vaccination supports our findings of low specificity for CDV using POCT-1 (17%) [25]. Another study that included both healthy client-owned and SPF dogs, reported high specificity for CDV with POCT-1 (87.5%) [24], but when considering only the client-owned dogs, specificity was notably lower (48.8%). Meazzi et al. reported good performance of POCT-1 for CDV (specificity of 87.2%), based on serum samples submitted for vaccination titer analysis, although the health status of the dogs was not specified [26]. Further studies on the performance of all POCTs in healthy dogs due to triennial vaccination and in dogs following completion of their puppy immunization series are required. Nevertheless, test reliability should also be ensured in dogs with chronic disease.

Virus neutralization tests detect neutralizing antibodies. IFAs and ELISAs—the test principle related to those of the POCTs—detect all binding antibodies, including non-neutralizing antibodies such as opsonizing or complement-activating antibodies [48,49]. These differences in antibody detection might have led to false-positive results (see Figure 4) and lower specificity when comparing POCTs to VN. However, neutralizing antibodies correlate well with protection, as demonstrated in several challenge studies with field and vaccine strain viruses [50,51]. Whether non-neutralizing antibodies also confer protection against CPV-2, CDV and CAV needs to be addressed in further studies.

Detection of MDA is not commonly applied, but can be useful for the determination of the optimal timepoint for puppy immunization in nomograph studies [52]. Virus neutralization is the assay of choice, as it provides exact endpoint titers and evaluates antibodies for their biological function. Our data support that POCT-1 also might be helpful for determining the appropriate vaccination timing in puppies in environments with high infection pressure of CPV-2, even though the manufacturer of POCT-1 does not recommend using the test for puppies younger than 20 weeks of age. POCT-2 and POCT-3 were not reliable in detecting MDAs, even though their manufacturers did not specify age-related limitations for their use. The inability to detect MDAs that interfere with vaccination could result in failure of puppy immunization [53].

Regarding practicability, POCT-2 and POCT-3 can be considered as true POCTs with only two steps taking only a few minutes to conduct, whereas POCT-1 requires at least 21 min of hands-on time due to its multiple test steps. A device for POCT-1, the RoboComb (Biogal Galed Labs), can reduce time spent by personnel, but was not included in this study.

Potential limitations of our study include performing POCTs in singlicate and using VN rather than hemagglutination inhibition (HI) as the reference method for CPV-2 testing. However, both VN and HI assess the biological functionality of antibodies and are strongly correlated with protective immunity [54]. Moreover, the use of VN facilitates direct comparison with previous studies from Germany, which also employed this method [23,27,28].

## 5. Conclusions

In light of the current vaccination recommendations, our findings suggest that improvements to all POCTs for detecting antibodies against CDV and CAV are warranted. At present the use of these POCTs for CDV and CAV may therefore be limited, with VN remaining the preferred method.

Since CDV and CAV vaccines are only available in combination with CPV-2, testing solely for CPV-2 antibodies is of limited clinical value—except in environments with high CPV pressure, where the infection risk for puppies is elevated. In such cases, POC testing can be generally helpful, and POCT-1 may offer additional value in detecting MDA.

Further evaluation of POCTs is warranted in healthy adult dogs at the time of scheduled triennial revaccination, as well as in subadult dogs following completion of puppy immunization. Further valuable directions for future research include confirming the presence of non-neutralizing antibodies using ELISA in addition to IFA, particularly relevant given that the POCTs are ELISA-based, and evaluating the potential protective role of non-neutralizing antibodies in dogs through virus challenge studies.

## Figures and Tables

**Table 1:** Signalment and health status of client-owned dogs (*n* = 200).

Characteristics	Category	Number of Dogs (*n*)	Years
Sex	Female	87	
	Male	113	
Neutering status	Intact	99	
	Neutered	101	
Breed	Purebred	159	
	Mixed breed	41	
Age	Range		0.2–17.2
	Median ± SD *		7.7 ± 4.3
Health status	Healthy or chronically ill	76	
	Acutely ill	124	

* SD, standard deviation.

**Table 2:** Characteristics of point-of-care tests (POCTs).

	POCT-1 Canine VacciCheck^®^ *	POCT-2 VCheck CPV/CDV/CAV Ab **	POCT-3 Fassisi CantiCheck Plus ***
Method	Enzyme-linked immunosorbent dot blot assay	Lateral flow test	Lateral flow test
Antibodies	CPV, CDV, CAV-1	CPV, CDV, CAV-1 & CAV-2	CPV, CDV, CAV-2
Readout	Semiquantitative, visual	Semiquantitative, analyzer	Qualitative, visual
Sample volume	1 × 5 μL serum/plasma or 10 μL whole blood	3 × 5 μL serum/plasma	3 × 20 μL serum, plasma or whole blood
Storage	2–8 °C, warm-up to room temperature 1 h prior to testing required	Room temperature	Room temperature
Incubation time	21 min	10 min	10 min

* Biogal Galed Labs, Kibbutz Galed, Israel; approved by the United States Department for Agriculture (USDA, Washington, DC, USA), Canadian Food Inspection Agency (CFIA, Ottawa, ON, Canada), and other regulatory authorities; ** BioNote Inc., Hwaseong-si, Republic of Korea; *** Fassisi^®^, Gesellschaft für Veterinärdiagnostik und Umweltanalysen mbH, Göttingen, Germany; CPV, canine parvovirus; CDV, canine distemper virus; CAV, canine adenovirus.

**Table 3:** Sensitivity, specificity, positive and negative predictive value and overall accuracy for the POCTs when compared to virus neutralization (VN)—for CPV-2.

POCT	Sensitivity % (95% CI %)	True Positive/ False Negative	Specificity % (95% CI %)	True Negative/ False Positive	Positive Predictive Value % (95% CI %)	Negative Predictive Value % (95% CI %)	Overall Accuracy % (95% CI %)
All dog sera (*n* = 240 *)							
POCT-1	100 (98.1–100)	194/0	100 (92.1–100)	45/0	100 (98.1–100)	100 (92.1–100)	100 (98.5–100)
POCT-2	93.9 (89.6–96.5)	183/12	97.8 (88.4–99.9)	44/1	99.5 (97.0–100.0)	78.6 (66.2–87.3)	94.6 (90.9–97.1)
POCT-3	79.0 (72.7–84.1)	154/41	100 (92.1–100)	45/0	100 (97.6–100)	52.3 (41.9–62.6)	82.92 (77.6–87.5)
Subgroup client-owned dogs (*n* = 200 **)							
POCT-1	100 (98.1–100)	194/0	100 (56.6–100)	5/0	100 (98.1–100)	100 (56.6–100)	100 (98.2–100)
POCT-2	93.9 (89.6–96.5)	183/12	80.0 (37.6–99.0)	4/1	99.5 (97.0–100.0)	25.0 (10.2–49.5)	93.50 (89.1–96.5)
POCT-3	79.0 (72.7–84.1)	154/41	100 (56.6–100)	5/0	100 (97.6–100)	10.9 (4.7–23.0)	79.50 (73.2–84.9)
Healthy or chronically ill (*n* = 76 ***)							
POCT-1	100 (94.9–100)	(72/0)	100 (43.9–100)	(3/0)	100 (94.9–100)	100 (43.9–100)	100 (95.2–100)
POCT-2	97.3 (90.6–99.5)	(71/2)	66.7 (11.9–98.3)	(2/1)	98.6 (92.5–99.9)	50.0 (8.9–91.1)	97.3 (90.7–99.7)
POCT-3	79.4 (68.8–87.1)	(58/15)	100 (43.9–100)	(3/0)	100 (93.8–100)	16.7 (5.8–39.2)	80.3 (69.5–88.5)
Acutely ill (*n* = 124)							
POCT-1	100 (97.0–100)	(122/0)	100 (17.8–100)	(2/0)	100 (97.0–100)	100 (17.8–100)	100 (97.1–100)
POCT-2	91.8 (85.6–95.5)	(112/10)	100 (17.8–100)	(2/0)	100 (97.0–100)	16.7 (3.0–44.8)	91.9 (85.7–96.1)
POCT-3	78.7 (70.6–85.0)	(96/26)	100 (17.8–100)	(2/0)	100 (97.0–100)	7.1 (1.3–22.7)	79.3 (70.8–85.8)
Subgroup specific pathogen-free (SPF) and specified antibody-free dogs (*n* = 40)							
POCT-1	n.a.	0/0	100 (91.2–100)	(40/0)	n.a.	100 (91.2–100)	n.a.
POCT-2	n.a.	0/0	100 (91.2–100)	(40/0)	n.a.	100 (91.2–100)	n.a.
POCT-3	n.a.	0/0	100 (91.2–100)	(40/0)	n.a.	100 (91.2–100)	n.a.

CI, confidence interval; * POCT-1 *n* = 239, ** POCT-1 *n* = 199, *** POCT-1 *n* = 75; n.a., not applicable.

**Table 4:** Sensitivity, specificity, positive and negative predictive value and overall accuracy for the POCTs when compared to VN—for CDV.

POCT	Sensitivity % (95% CI %)	True Positive/ False Negative	Specificity % (95% CI %)	True Negative/ False Positive	Positive Predictive Value % (95% CI %)	Negative Predictive Value % (95% CI %)	Overall Accuracy % (95% CI %)
All dog sera (*n* = 240 *)							
POCT-1	98.7 (95.4–99.8)	152/2	43.5 (33.5–54.1)	37/48	76.0 (69.6–81.4)	94.9 (83.1–99.1)	79.1 (73.4–84.1)
POCT-2	98.7 (95.4–99.8)	153/2	42.4 (32.4–53.0)	36/49	75.7 (69.4–81.1)	94.7 (82.7–99.1)	78.8 (73.0–83.8)
POCT-3	17.4 (12.3–24.2)	27/128	94.1 (87.0–97.5)	80/5	84.4 (68.3–93.1)	38.46 (32.1–45.2)	44.6 (38.2–51.1)
Subgroup client-owned dogs (*n* = 200 **)							
POCT-1	98.7 (95.4–99.8)	152/2	13.3 (62.6–26.2)	6/39	79.6 (73.3–84.7)	75.0 (40.9–95.6)	79.4 (73.1–84.8)
POCT-2	98.7 (95.4–99.8)	153/2	17.8 (9.3–31.3)	8/37	80.5 (74.3–85.5)	80.0 (49.0–96.5)	80.5 (74.3–85.8)
POCT-3	17.4 (12.3–24.2)	27/128	93.3 (82.1–97.7)	42/3	90.0 (74.4–96.5)	24.7 (8.8–31.7)	34.5 (27.9–41.5)
Healthy or chronically ill (*n* = 76 ***)							
POCT-1	96.7 (88.8–99.4)	58/3	7.1 (0.4–31.5)	1/13	81.9 (71.5–89.1)	33.3 (1.7–88.2)	80.0 (69.2–88.4)
POCT-2	98.4 (91.4–99.9)	61/1	7.1 (0.4–31.5)	1/13	82.4 (72.2–89.4)	50.0 (2.6–97.4)	81.6 (71.0–89.6)
POCT-3	19.4 (11.4–30.9)	12/50	85.7 (60.1–97.5)	12/2	85.7 (60.1–97.5)	19.4 (11.4–30.9)	31.6 (21.4–43.3)
Acutely ill (*n* = 124)							
POCT-1	100 (96.0–100)	93/0	16.1 (7.1–32.6)	5/26	78.2 (69.9–84.6)	100 (56.6–100)	79.0 (70.8–85.8)
POCT-2	98.9 (94.2–99.9)	92/1	22.6 (11.4–39.8)	7/24	79.3 (71.1–85.7)	87.5 (52.9–99.4)	79.8 (71.7–86.5)
POCT-3	16.1 (10.0–24.9)	15/78	96.8 (83.8–99.8)	30/1	93.8 (71.7–99.7)	27.8 (20.2–36.9)	36.3 (27.9–45.4)
Subgroup specific pathogen-free (SPF) and specified antibody-free dogs (*n* = 40)							
POCT-1	n.a.	0/0	77.5 (62.5–87.7)	31/9	0 (0–30.0)	100 (89.0–100)	n.a.
POCT-2	n.a.	0/0	70.0 (54.6–81.9)	28/12	0 (0–24.25)	100 (87.9–100)	n.a.
POCT-3	n.a.	0/0	95.0 (83.5–99.1)	38/2	0 (0–82.23)	100 (0.82–100)	n.a.

* POCT-1 *n* = 239, ** POCT-1 *n* = 199, *** POCT-1 *n* = 75., n.a. not applicable

**Table 5:** Sensitivity, specificity, positive and negative predictive value and overall accuracy for the POCTs when compared to VN—for CAV.

POCT	Sensitivity % (95% CI %)	True Positive/ False Negative	Specificity % (95% CI %)	True Negative/ False Positive	Positive Predictive Value % (95% CI %)	Negative Predictive Value % (95% CI %)	Overall Accuracy % (95% CI %)
All dog sera (*n* = 240 *)							
POCT-1	99.4 (96.6–100)	162/1	55.3 (44.1–65.9)	42/34	82.7 (76.7–87.3)	97.7 (87.9–99.9)	85.4 (80.2–89.6)
POCT-2	90.8 (85.4–94.3)	148/15	79.2 (68.9–86.8)	61/16	90.2 (84.7–93.9)	80.3 (70.0–87.7)	87.1 (82.2–91.1)
POCT-3	33.1 (26.4–40.7)	54/109	84.4 (74.7–90.9)	65/12	81.8 (70.9–89.3)	37.4 (30.5–44.7)	43.6 (43.1–56.1)
Subgroup client-owned dogs (*n* = 200 **)							
POCT-1	99.4 (96.6–100)	162/1	8.3 (2.9–21.8)	3/33	83.1 (77.2–87.7)	75.0 (30.1–98.7)	82.9 (77.0–87.9)
POCT-2	90.8 (85.4–94.3)	148/15	56.8 (40.9–71.3)	21/16	90.2 (84.7–93.9)	58.3 (42.2–72.9)	84.5 (78.7–89.2)
POCT-3	33.1 (26.4–40.7)	54/109	81.1 (65.8–90.5)	30/7	88.5 (78.2–94.3)	21.6 (15.6–29.1)	42 (35.1–49.2)
Healthy or chronically ill (*n* = 76 ***)							
POCT-1	100 (94.4–100)	65/0	0 (0–27.8)	0/10	86.7 (77.2–92.6)	n.a.	86.7 (76.8–93.4)
POCT-2	93.9 (85.2–97.6)	61/4	36.4 (15.2–64.6)	4/7	89.7 (80.2–94.9)	50.0 (21.5–78.5)	85.5 (75.6–92.6)
POCT-3	38.5 (27.6–50.6)	25/40	81.8 (52.3–96.8)	9/2	92.6 (76.6–98.7)	18.4 (9.98–31.4)	44.7 (33.3–56.6)
Acutely ill (*n* = 124)							
POCT-1	99.0 (94.4–100)	97/1	11.5 (4.0–29.0)	3/23	80.8 (72.9–86.9)	75.0 (30.1–98.7)	80.7 (72.6–87.2)
POCT-2	88.8 (81.0–93.6)	87/11	65.4 (46.2–80.6)	17/9	90.6 (83.1–95.0)	60.7 (42.4–76.4)	83.9 (76.2–89.9)
POCT-3	69.4 (59.7–77.6)	29/69	80.8 (62.1–91.5)	21/5	93.2 (85.0–97.0)	41.2 (28.8–54.8)	71.8 (63.0–79.5)
Subgroup specific pathogen-free (SPF), specified antibody-free dogs (*n* = 40)							
POCT-1	n.a.	0/0	97.5 (87.1–99.9)	39/1	0 (0–94.9)	100 (91.0–100)	n.a.
POCT-2	n.a.	0/0	100 (91.2–100)	40/0	n.a.	100 (91.2–100)	n.a.
POCT-3	n.a.	0/0	87.5 (73.9–94.5)	35/5	0 (0–43.5)	100 (90.1–100)	n.a.

* POCT-1 *n* = 239, ** POCT-1 *n* = 199, *** POCT-1 *n* = 75, n.a. not applicable.

**Table 6:** Sensitivity, specificity, positive and negative predictive value and overall accuracy for the POCTs when compared to VN for SPF dogs with maternally derived antibodies (MDA) (*n* = 20).

Virus	POCT	Sensitivity % (95% CI %)	True Positive/ False Negative	Specificity % (95% CI %)	True Negative/ False Positive	Positive Predictive Value % (95% CI %)	Negative Predictive Value % (95% CI %)	Overall Accuracy % (95% CI %)
CPV-2	POCT-1 *	95 (76.4–99.7)	19/1	n.a.	0/0	100 (83.2–100)	0 (0–94.9)	n.a.
	POCT-2	30 (14.6–51.9)	6/14	n.a.	0/0	100 (61.0–100)	0 (0–21.5)	n.a.
	POCT-3	0 (0–16.1)	0/20	n.a.	0/0	n.a.	0 (0–16.1)	n.a.
CDV	POCT-1 *	80.0 (37.6–99.0)	4/1	26.7 (10.9–52.0)	4/11	26.7 (10.9–52.0)	80 (37.6–99.0)	40.0 (19.1–64.0)
	POCT-2	60.0 (23.1–92.9)	3/2	40.0 (19.8–64.3)	6/9	25.0 (8.9–53.2)	75 (40.9–95.6)	45.0 (23.1–68.5)
	POCT-3	0 (0–43.5)	0/5	100 (79.6–100)	15/0	n.a.	75 (53.1–88.8)	75.0 (50.9–91.3)
CAV-1	POCT-1 *	92.3 (66.7–99.6)	12/1	14.3 (0.7–51.3)	1/6	66.7 (43.8–83.7)	50 (25.7–97.4)	65.0 (40.8–84.6)
	POCT-2	69.2 (42.4–87.3)	9/4	85.7 (48.7–99.3)	6/1	90 (59.6–99.5)	60 (31.3–83.2)	75.0 (50.9–91.3)
	POCT-3	0 (0–22.8)	0/13	100 (64.6–100)	7/0	n.a.	35 (18.1–56.7)	35.0 (15.4–59.2)

* Manufacturer does not recommend its use in dogs younger than 20 weeks of age; n.a. not applicable

**Table 7:** Degree of agreement between POCTs determined with Kappa coefficient regarding test results of all dogs (*n* = 240, SPF with maternally derived antibodies excluded).

Virus	Assay	Cohen’s Kappa (CI 95%)	Agreement
CPV-2	POCT-1 vs. POCT-2	0.72 (0.62–0.82)	Substantial
	POCT-1 vs. POCT-3	0.48 (0.38–0.57)	Moderate
	POCT-2 vs. POCT-3	0.66 (0.57–0.76)	Substantial
CDV	POCT-1 vs. POCT-2	0.54 (0.41–0.68)	Moderate
	POCT-1 vs. POCT-3	0.04 (0.01–0.07)	None/slight
	POCT-2 vs. POCT-3	0.05 (0.02–0.08)	None/slight
CAV	POCT-1 vs. POCT-2	0.60 (0.50–0.71)	Moderate
	POCT-1 vs. POCT-3	0.06 (0.01–0.12)	None/slight
	POCT-2 vs. POCT-3	0.06 (0.02–0.15)	None/slight

**Table 8:** Vaccination recommendation based on the test results for all dogs eligible for vaccination (*n* = 154, acutely ill dogs excluded); values for client-owned dogs only in brackets; if one or more of the three antibody titers is deemed insufficient for protection, the dog receives a recommendation for vaccination.

Test	Virus	Positive Result	Negative Result	Vaccination Recommended	
				Yes	No
VN	CPV-2	110	44	85 (29)	69 (65)
	CDV	80	74		
	CAV	93	61		
POCT-1 *	CPV-2	69	31	35 (5)	66 (66)
	CDV	76	24		
	CAV	71	29		
POCT-2	CPV-2	93	61	77 (18)	77 (76)
	CDV	113	41		
	CAV	92	62		
POCT-3	CPV-2	67	87	142 (82)	12 (12)
	CDV	21	133		
	CAV	35	119		

* POCT-1 *n* = 101 (one test result invalid), dogs < 20 weeks of age excluded, since the manufacturer recommends titer testing starting at 20 weeks of age.

## Data Availability

All relevant data are contained within the article.

## Appendix A

**Table A1:** Interferences specified by the POCT manufacturers, which may compromise the interpretation of test results.

Serum Indices	POCT-1	POCT-2	POCT-3
Triglycerides (mg/dL)	No interferences	CPV, CDV: 900 CAV: 1000	Testing is not recommended in case of visible lipemia, hemolysis or hyperbilirubinemia
Hemoglobin (mg/dL)	Hemolysis causes a dark background with indistinct test spots	10,000	
Bilirubin (mg/dL)	No interferences	CPV, CDV: 5 CAV: 15	

**Table A2:** Range of antibody titers and number of results for each titer level detected by virus neutralization (VN) for canine parvovirus type 2 (CPV-2), canine distemper virus (CDV) and canine adenovirus type 1 (CAV-1) in client-owned dogs. The reciprocal value of the highest dilution, at which no cytopathic effect was observed, corresponds to the antibody titer.

Titer	Number of Results for Each Titer Level		
	CPV-2	CDV	CAV-1
<10	5	45	37
10	0	25	16
20	0	31	31
40	3	45	28
80	4	27	29
160	19	17	29
320	38	7	15
640	43	3	8
1280	48	0	4
2560	25	0	3
5120	13	0	0
10,240	2	0	0

**Table A3:** Results for the detection of antibodies against CPV-2, detected by VN and three different point-of-care tests (POCTs) for all dogs (*n* = 260); antibody titers reflect the reciprocal of the last serum dilution not showing positive fluorescence in indirect IFA after conduction of VN.

Sample ID	VN		POCT-1			POCT-2		POCT-3
1	+	320	+	S4	160	+	120	−
2	+	640	+	S5	320	+	1280	+
3	+	320	+	S5	320	+	320	+
4	+	1280	+	S5	320	+	640	+
5	+	320	+	S5	320	+	320	+
6	+	640	+	S5	320	+	1280	+
7	+	160	+	S4	160	+	160	+
8	+	160	+	S4	160	+	160	+
9	+	160	+	S4	160	+	80	−
10	+	160	+	S6	≥640	+	1280	+
11	+	80	+	S2	40	+	120	−
12	+	640	+	S5	320	+	320	+
13	+	160	+	S4	160	+	240	+
14	+	320	+	S5	320	+	240	+
15	+	640	+	S6	≥640	+	640	+
16	+	320	+	S5	320	+	640	+
17	+	640	+	S5	320	+	640	+
18	+	320	+	S4	160	+	240	+
19	+	640	+	S5	320	+	320	+
20	+	160	+	S4	160	+	160	+
21	+	5120	+	S6	≥640	+	1280	+
22	+	320	+	S4	160	+	160	+
23	+	1280	+	S6	≥640	+	1280	+
24	+	1280	+	S6	≥640	+	1280	+
25	+	1280	+	S6	≥640	+	1280	+
26	+	320	+	S4	160	+	160	+
27	+	5120	+	S6	≥640	+	1280	+
28	+	640	+	S5	320	+	320	+
29	+	320	invalid			+	80	+
30	+	320	+	S4	160	+	120	−
31	+	1280	+	S5	320	+	320	+
32	+	640	+	S4	160	+	240	+
33	+	640	+	S5	320	+	240	+
34	+	640	+	S5	320	+	320	+
35	+	320	+	S4	160	+	80	−
36	+	1280	+	S5	320	+	1280	+
37	+	1280	+	S5	320	+	1280	+
38	+	1280	+	S5	320	+	320	+
39	+	160	+	S4	160	−	20	−
40	+	640	+	S3	80	+	240	−
41	+	2560	+	S5	320	+	640	+
42	+	320	+	S4	160	+	120	−
43	+	320	+	S4	160	+	160	−
44	+	≥10,240	+	S5	320	+	1280	−
45	+	1280	+	S5	320	+	1280	+
46	+	5120	+	S5	320	+	1280	+
47	+	320	+	S4	160	+	120	−
48	+	1280	+	S5	320	+	1280	−
49	+	2560	+	S5	320	+	1280	+
50	+	160	+	S3	80	+	120	−
51	+	1280	+	S5	320	+	1280	+
52	+	1280	+	S5	320	+	1280	+
53	+	640	+	S5	320	+	1280	+
54	+	1280	+	S5	320	+	640	+
55	+	640	+	S5	320	+	240	−
56	+	640	+	S5	320	+	320	+
57	+	1280	+	S5	320	+	640	+
58	+	320	+	S4	160	+	320	+
59	+	320	+	S4	160	+	240	−
60	+	640	+	S4	160	+	240	+
61	+	640	+	S4	160	+	320	+
62	+	1280	+	S5	320	+	240	+
63	+	320	+	S4	160	+	320	−
64	+	2560	+	S5	320	+	640	+
65	+	320	+	S3	80	+	160	−
66	+	640	+	S5	320	+	320	+
67	+	80	+	S3	80	+	80	+
68	+	640	+	S4	160	+	240	+
69	+	1280	+	S5	320	+	320	+
70	+	640	+	S4	160	+	160	−
71	+	320	+	S4	160	−	40	−
72	+	1280	+	S5	320	+	640	+
73	+	320	+	S3	80	−	20	−
74	+	2560	+	S5	320	+	1280	+
75	+	160	+	S4	160	+	120	−
76	+	80	+	S3	80	−	20	−
77	+	640	+	S4	160	+	240	+
78	+	160	+	S4	160	+	240	+
79	+	320	+	S4	160	+	240	+
80	+	320	+	S5	320	+	240	+
81	+	5120	+	S5	320	+	1280	+
82	+	5120	+	S5	320	+	1280	+
83	+	1280	+	S6	≥640	+	640	+
84	+	640	+	S5	320	+	640	−
85	+	1280	+	S5	320	+	1280	+
86	−	<10	−	S0	−	−	40	−
87	+	640	+	S4	160	+	240	+
88	+	160	+	S4	160	+	120	−
89	+	640	+	S4	160	+	240	+
90	+	160	+	S5	320	−	20	+
91	+	320	+	S4	160	+	240	+
92	+	2560	+	S6	≥640	+	1280	+
93	+	≥10,240	+	S6	≥640	+	1280	+
94	+	160	+	S4	160	+	80	+
95	+	640	+	S4	160	+	240	+
96	+	1280	+	S5	320	+	640	+
97	+	640	+	S5	320	+	240	+
98	+	1280	+	S5	320	+	320	+
99	+	1280	+	S5	320	+	640	+
100	+	2560	+	S6	≥640	+	1280	+
101	−	<10	−	S1	−	−	≤10	−
102	+	320	+	S5	320	+	320	+
103	+	320	+	S5	320	+	640	+
104	+	1280	+	S5	320	+	640	+
105	+	160	+	S5	320	+	160	+
106	+	2560	+	S5	320	+	1280	+
107	+	320	+	S4	160	−	40	+
108	+	40	+	S4	160	+	160	−
109	+	320	+	S5	320	+	240	+
110	+	320	+	S5	320	+	240	+
111	+	1280	+	S5	320	+	320	+
112	+	1280	+	S5	320	+	640	+
113	+	2560	+	S5	320	+	1280	+
114	+	5120	+	S5	320	+	1280	+
115	+	1280	+	S5	320	+	640	+
116	+	320	+	S4	160	+	160	+
117	+	160	+	S4	160	+	160	−
118	+	320	+	S5	320	+	240	+
119	+	640	+	S5	320	+	320	−
120	+	320	+	S5	320	+	240	+
121	+	1280	+	S6	≥640	+	320	+
122	+	2560	+	S5	320	+	1280	+
123	+	1280	+	S5	320	+	1280	+
124	−	<10	−	S1	−	+	240	−
125	−	<10	−	S1	−	−	20	−
126	+	640	+	S5	320	+	240	+
127	+	1280	+	S6	≥640	+	1280	+
128	+	640	+	S4	160	+	320	+
129	+	2560	+	S5	320	+	320	+
130	+	1280	+	S6	≥640	+	1280	+
131	+	2560	+	S5	320	+	640	+
132	+	640	+	S4	160	+	240	+
133	+	160	+	S4	160	+	120	+
134	+	1280	+	S5	320	+	320	+
135	+	2560	+	S6	≥640	+	1280	+
136	+	640	+	S6	≥640	+	240	−
137	+	320	+	S5	320	+	160	+
138	+	1280	+	S6	≥640	+	240	+
139	+	5120	+	S6	≥640	+	≥1280	+
140	+	320	+	S4	160	+	160	+
141	+	5120	+	S6	≥640	+	1280	+
142	+	2560	+	S6	≥640	+	1280	+
143	+	640	+	S4	160	+	120	−
144	+	2560	+	S6	≥640	+	640	+
145	+	2560	+	S6	≥640	+	640	+
146	+	1280	+	S6	≥640	+	1280	+
147	+	2560	+	S6	≥640	+	1280	+
148	+	640	+	S4	160	+	120	+
149	+	1280	+	S5	320	+	240	+
150	+	2560	+	S6	≥640	+	320	+
151	+	640	+	S4	160	+	160	+
152	+	320	+	S4	160	+	160	+
153	+	2560	+	S6	≥640	+	320	+
154	+	1280	+	S6	≥640	+	240	−
155	+	2560	+	S6	≥640	+	1280	+
156	+	1280	+	S6	≥640	+	240	+
157	+	160	+	S3	80	−	40	−
158	+	5120	+	S6	≥640	+	1280	+
159	+	2560	+	S6	≥640	+	1280	+
160	+	640	+	S4	160	+	160	+
161	+	5120	+	S6	≥640	+	1280	+
162	+	40	+	S2	40	−	20	−
163	+	320	+	S4	160	−	20	−
164	+	160	+	S6	≥640	+	640	+
165	+	1280	+	S5	320	+	240	+
166	+	1280	+	S5	320	+	320	+
167	+	320	+	S4	160	+	120	−
168	+	1280	+	S6	≥640	+	1280	+
169	+	1280	+	S6	≥640	+	1280	+
170	+	2560	+	S6	≥640	+	640	+
171	+	1280	+	S5	320	+	240	+
172	−	<10	−	S1	−	−	≤10	−
173	+	2560	+	S6	≥640	+	320	+
174	+	640	+	S5	320	+	240	+
175	+	640	+	S5	320	+	240	+
176	+	5120	+	S6	≥640	+	1280	+
177	+	1280	+	S6	≥640	+	320	+
178	+	640	+	S4	160	+	240	+
179	+	1280	+	S5	320	+	320	+
180	+	1280	+	S5	320	+	320	+
181	+	80	+	S3	80	+	120	−
182	+	1280	+	S5	320	+	320	+
183	+	40	+	S3	80	−	40	−
184	+	640	+	S5	320	+	240	+
185	+	320	+	S4	160	−	20	−
186	+	2560	+	S6	≥640	+	1280	+
187	+	5120	+	S6	≥640	+	1280	−
188	+	2560	+	S5	320	+	1280	+
189	+	640	+	S4	160	+	240	+
190	+	320	+	S4	160	+	160	+
191	+	1280	+	S5	320	+	640	+
192	+	640	+	S5	320	+	320	−
193	+	1280	+	S5	320	+	240	+
194	+	5120	+	S6	≥640	+	1280	+
195	+	1280	+	S4	160	−	20	−
196	+	160	+	S3	80	+	120	−
197	+	640	+	S5	320	+	640	+
198	+	640	+	S4	160	+	320	+
199	+	2560	+	S5	320	+	640	+
200	+	640	+	S4	160	+	320	+
201	−	<10	−	S0	−	−	≤10	−
202	−	<10	−	S0	−	−	≤10	−
203	−	<10	−	S0	−	−	≤10	−
204	−	<10	−	S0	−	−	≤10	−
205	−	<10	−	S0	−	−	≤10	−
206	−	<10	−	S0	−	−	≤10	−
207	−	<10	−	S0	−	−	≤10	−
208	−	<10	−	S0	−	−	≤10	−
209	−	<10	−	S0	−	−	≤10	−
210	−	<10	−	S0	−	−	≤10	−
211	−	<10	−	S0	−	−	≤10	−
212	−	<10	−	S0	−	−	≤10	−
213	−	<10	−	S0	−	−	≤10	−
214	−	<10	−	S0	−	−	≤10	−
215	−	<10	−	S0	−	−	≤10	−
216	−	<10	−	S0	−	−	≤10	−
217	−	<10	−	S0	−	−	≤10	−
218	−	<10	−	S0	−	−	≤10	−
219	−	<10	−	S0	−	−	≤10	−
220	−	<10	−	S0	−	−	≤10	−
221	−	<10	−	S0	−	−	≤10	−
222	−	<10	−	S0	−	−	≤10	−
223	−	<10	−	S0	−	−	≤10	−
224	−	<10	−	S0	−	−	≤10	−
225	−	<10	−	S1	−	−	≤10	−
226	−	<10	−	S0	−	−	≤10	−
227	−	<10	−	S0	−	−	≤10	−
228	−	<10	−	S0	−	−	≤10	−
229	−	<10	−	S1	−	−	≤10	−
230	−	<10	−	S0	−	−	≤10	−
231	−	<10	−	S0	−	−	≤10	−
232	−	<10	−	S0	−	−	≤10	−
233	−	<10	−	S0	−	−	≤10	−
234	−	<10	−	S0	−	−	≤10	−
235	−	<10	−	S0	−	−	≤10	−
236	−	<10	−	S0	−	−	≤10	−
237	−	<10	−	S0	−	−	≤10	−
238	−	<10	−	S0	−	−	≤10	−
239	−	<10	−	S0	−	−	≤10	−
240	−	<10	−	S0	−	−	≤10	−
241	+	80	+	S2	40	+	80	−
242	+	80	+	S2	40	+	80	−
243	+	80	+	S2	40	−	40	−
244	+	20	+	S2	40	+	80	−
245	+	80	+	S2	40	−	40	−
246	+	160	+	S3	80	+	80	−
247	+	20	+	S3	80	−	20	−
248	+	20	+	S3	80	−	20	−
249	+	10	+	S3	80	−	20	−
250	+	10	−	S1	−	−	10	−
251	+	10	+	S2	40	−	20	−
252	+	40	+	S3	80	−	40	−
253	+	20	+	S2	40	−	40	−
254	+	20	+	S2	40	−	20	−
255	+	80	+	S3	80	−	40	−
256	+	10	+	S2	40	−	40	−
257	+	80	+	S4	160	+	80	−
258	+	20	+	S3	80	−	40	−
259	+	20	+	S2	40	−	20	−
260	+	80	+	S3	80	+	80	−

+, positive; − negative; S0–S6, scores corresponding to the CombScale (Biogal Galed Labs) of POCT-1.

**Table A4:** Results for the detection of antibodies against CDV, detected by VN and three different POCTs for all dogs (*n* = 260); results by immunofluorescence assay (IFA), where POCTs showed false-positive results. The antibody titer is defined as the reciprocal of the highest serum dilution at which no cytopathic effect is observed in VN. In the IFA, the antibody titer corresponds to the reciprocal of the last serum dilution that shows positive fluorescence.

Sample ID	VN		POCT-1			POCT-2		POCT-3	IFA	
1	+	20	+	S4	64	+	96	+		
2	+	160	+	S4	64	+	128	−		
3	+	80	+	S3	32	+	128	+		
4	+	80	+	S4	64	+	256	+		
5	+	20	+	S3	32	+	128	−		
6	+	160	+	S4	64	+	512	+		
7	−	<10	+	S4	64	+	32	−	−	<1:80
8	−	<10	+	S4	64	+	32	−	−	<1:80
9	+	20	−	S1	−	+	64	−		
10	+	80	+	S5	≥128	+	256	−		
11	−	<10	+	S4	64	+	48	+	−	<1:80
12	+	80	+	S5	≥128	+	512	−		
13	+	40	+	S3	32	+	64	−		
14	+	160	+	S4	64	+	256	−		
15	+	320	+	S4	64	−	≤4	−		
16	+	80	+	S6	≥256	+	96	+		
17	+	20	+	S4	64	+	64	−		
18	+	40	+	S4	64	+	64	+		
19	+	40	+	S4	64	+	64	+		
20	−	<10	+	S4	64	+	48	−	−	<1:80
21	+	10	+	S2	16	+	48	−		
22	−	<10	+	S3	32	+	32	−	−	<1:80
23	+	40	+	S4	64	+	64	+		
24	−	<10	+	S5	≥128	+	48	+	+	160
25	+	320	+	S5	≥128	+	96	+		
26	+	40	+	S3	32	+	96	−		
27	+	80	+	S4	64	+	96	−		
28	+	80	+	S5	≥128	+	256	−		
29	+	40	invalid			+	96	−		
30	−	<10	−	S1	−	+	48	−	−	<1:80
31	+	40	+	S4	64	+	64	−		
32	−	<10	−	S1	−	+	48	−	−	<1:80
33	+	20	+	S3	32	+	128	−		
34	−	<10	+	S4	64	−	16	−	+	160
35	−	<10	+	S3	32	+	48	−	−	<1:80
36	+	80	+	S5	≥128	+	256	−		
37	+	160	+	S5	≥128	+	128	−		
38	+	80	+	S4	64	+	256	+		
39	+	80	+	S3	32	+	48	−		
40	+	80	+	S3	32	+	96	−		
41	−	<10	+	S3	32	+	128	−	−	<1:80
42	+	10	+	S3	32	−	16	−		
43	+	20	+	S2	16	+	48	−		
44	+	640	+	S5	≥128	+	512	−		
45	−	<10	+	S5	≥128	+	64	−	−	<1:80
46	+	10	+	S3	32	+	32	−		
47	−	<10	+	S4	64	+	128	−	+	80
48	+	160	+	S4	64	+	32	−		
49	+	40	+	S5	≥128	+	96	−		
50	+	10	+	S2	16	+	96	−		
51	+	40	+	S5	≥128	+	96	−		
52	+	20	+	S3	32	+	96	−		
53	+	10	+	S3	32	+	48	−		
54	−	<10	+	S5	≥128	+	96	−	+	≥320
55	+	20	+	S3	32	+	48	−		
56	+	40	+	S4	64	+	96	−		
57	+	80	+	S5	≥128	+	64	−		
58	+	40	+	S2	16	+	96	−		
59	+	10	−	S1	−	+	64	−		
60	+	40	+	S3	32	+	96	+		
61	+	160	+	S4	64	+	256	−		
62	+	10	+	S2	16	+	32	−		
63	+	20	+	S3	32	+	96	−		
64	+	80	+	S4	64	+	256	−		
65	+	20	+	S2	16	+	96	−		
66	+	40	+	S4	64	+	64	−		
67	+	40	+	S2	16	+	96	−		
68	+	40	+	S3	32	+	48	−		
69	+	20	+	S3	32	+	96	−		
70	+	160	+	S3	32	+	96	−		
71	+	10	+	S2	16	+	32	−		
72	+	20	+	S3	32	+	48	−		
73	+	40	+	S4	64	+	48	−		
74	+	80	+	S5	≥128	+	256	−		
75	+	40	+	S3	32	+	96	−		
76	+	40	+	S4	64	+	64	−		
77	+	80	+	S4	64	+	128	−		
78	−	<10	+	S3	32	+	32	−	+	160
79	+	40	+	S3	32	+	64	−		
80	+	160	+	S4	64	+	128	−		
81	+	160	+	S4	64	+	128	−		
82	+	40	+	S4	64	+	256	−		
83	−	<10	+	S2	16	+	48	−	−	<1:80
84	+	40	+	S3	32	+	128	+		
85	−	<10	+	S5	≥128	+	48	−	+	320
86	+	160	+	S5	≥128	+	96	−		
87	+	10	+	S4	64	+	48	−		
88	+	20	+	S3	32	+	48	−		
89	−	<10	+	S4	64	+	512	−	+	160
90	−	<10	+	S3	32	+	48	−	−	<1:80
91	−	<10	+	S3	32	+	48	−	−	<1:80
92	+	40	+	S5	≥128	+	64	−		
93	+	160	+	S5	≥128	+	128	−		
94	−	<10	−	S1	−	+	32	−	−	<1:80
95	+	10	+	S3	32	+	96	+		
96	+	10	+	S4	64	+	96	−		
97	+	10	+	S5	≥128	+	48	−		
98	+	10	+	S4	64	+	32	−		
99	+	40	+	S4	64	+	128	−		
100	−	<10	+	S5	≥128	+	48	−	+	≥320
101	−	<10	+	S2	16	+	32	−	+	160
102	+	20	+	S5	≥128	+	48	−		
103	+	20	+	S2	16	+	64	−		
104	−	<10	+	S4	64	+	48	−	+	80
105	+	40	+	S4	64	+	64	+		
106	+	20	+	S4	64	+	48	−		
107	−	<10	+	S3	32	−	16	−	−	<1:80
108	−	<10	+	S3	32	−	16	+	−	<1:80
109	+	40	+	S5	≥128	+	256	−		
110	+	320	+	S5	≥128	+	96	−		
111	+	80	+	S4	64	+	96	−		
112	+	10	+	S3	32	+	48	−		
113	+	160	+	S4	64	+	64	+		
114	+	640	+	S6	≥256	+	512	−		
115	−	<10	+	S6	≥256	+	64	−	+	320
116	+	320	+	S4	64	+	96	−		
117	+	80	+	S4	64	+	96	−		
118	+	160	+	S5	≥128	+	96	−		
119	+	320	+	S6	≥256	+	256	+		
120	+	20	+	S4	64	+	48	−		
121	+	320	+	S5	≥128	+	256	+		
122	+	640	+	S4	64	+	256	−		
123	+	320	+	S4	64	+	256	−		
124	+	80	+	S4	64	+	128	−		
125	−	<10	+	S6	≥256	+	64	−	+	≥320
126	−	<10	+	S3	32	+	48	−	+	80
127	−	<10	+	S3	32	+	512	−	+	80
128	+	40	+	S3	32	+	64	−		
129	−	<10	+	S3	32	+	48	−	+	80
130	−	<10	+	S2	16	−	16	−	−	<1:80
131	+	40	+	S6	≥256	+	96	−		
132	+	20	+	S4	64	+	48	−		
133	+	10	+	S3	32	+	128	+		
134	+	80	+	S4	64	+	128	+		
135	+	80	+	S5	≥128	+	128	+		
136	+	40	+	S4	64	+	48	−		
137	+	40	+	S5	≥128	+	96	−		
138	+	10	+	S3	32	+	64	−		
139	+	10	+	S4	64	+	48	−		
140	+	20	+	S4	64	+	64	−		
141	+	80	+	S5	≥128	+	64	−		
142	−	<10	+	S5	≥128	+	64	−	+	≥320
143	+	40	+	S4	64	+	48	+		
144	+	20	+	S3	32	+	96	−		
145	+	40	+	S4	64	+	96	−		
146	+	10	+	S5	≥128	+	48	−		
147	+	80	+	S3	32	+	64	−		
148	+	10	+	S3	32	+	48	+		
149	+	10	+	S3	32	+	48	−		
150	+	20	+	S5	≥128	+	128	−		
151	+	20	+	S4	64	+	96	+		
152	+	10	+	S4	64	+	48	−		
153	−	<10	+	S3	32	+	48	−	+	80
154	−	<10	+	S3	32	+	32	−	+	80
155	+	160	+	S4	64	+	512	−		
156	+	80	+	S5	≥128	+	48	−		
157	−	<10	+	S3	32	+	32	−	+	80
158	+	20	+	S5	≥128	+	128	−		
159	+	20	+	S5	≥128	+	96	−		
160	+	20	+	S4	64	+	96	−		
161	+	40	+	S4	64	+	48	−		
162	−	<10	−	S1	−	−	≤4	−		
163	+	40	+	S4	64	+	48	−		
164	+	40	+	S4	64	+	128	−		
165	−	<10	−	S1	−	−	16	−		
166	+	20	+	S4	64	+	512	+		
167	−	<10	+	S4	64	+	32	−	+	160
168	+	160	+	S5	≥128	+	96	−		
169	+	20	+	S5	≥128	+	64	−		
170	+	40	+	S5	≥128	+	64	−		
171	+	20	+	S4	64	+	48	+		
172	−	<10	−	S1	−	−	16	−		
173	+	10	+	S5	≥128	+	48	−		
174	+	20	+	S4	64	+	64	+		
175	+	40	+	S4	64	+	64	+		
176	+	40	+	S4	64	+	96	−		
177	+	10	+	S4	64	+	48	−		
178	+	20	+	S3	32	+	128	−		
179	+	160	+	S5	≥128	+	256	−		
180	+	40	+	S4	64	+	64	−		
181	+	40	+	S4	64	+	128	−		
182	−	<10	+	S5	≥128	+	96	−	+	≥320
183	+	10	+	S3	32	+	32	−		
184	+	40	+	S4	64	+	96	−		
185	+	10	+	S4	64	+	32	−		
186	+	40	+	S4	64	+	48	−		
187	+	80	+	S4	64	+	64	−		
188	+	40	+	S3	32	+	64	−		
189	+	20	+	S3	32	+	48	−		
190	−	<10	+	S3	32	+	128	−	−	<1:80
191	+	40	+	S4	64	+	64	−		
192	+	160	+	S5	≥128	+	128	−		
193	+	40	+	S5	≥128	+	96	−		
194	+	80	+	S4	64	+	64	−		
195	−	<10	+	S2	16	+	32	−	+	≥320
196	−	<10	+	S2	16	+	48	−	−	<1:80
197	+	80	+	S4	64	+	48	−		
198	+	40	+	S4	64	+	48	−		
199	+	80	+	S4	64	+	64	−		
200	−	<10	+	S3	32	−	16	−	−	<1:80
201	−	<10	−	S0	−	+	32	+	−	<1:80
202	−	<10	−	S0	−	+	32	+	−	<1:80
203	−	<10	−	S1	−	−	16	−		
204	−	<10	−	S0	−	−	16	−		
205	−	<10	−	S0	−	+	32	−	−	<1:80
206	−	<10	−	S0	−	+	32	−	−	<1:80
207	−	<10	−	S0	−	−	16	−		
208	−	<10	−	S0	−	−	16	−		
209	−	<10	−	S0	−	−	16	−		
210	−	<10	−	S0	−	+	48	−	−	<1:80
211	−	<10	−	S1	−	−	16	−		
212	−	<10	+	S2	16	+	32	−	−	<1:80
213	−	<10	+	S2	16	+	32	−	−	<1:80
214	−	<10	+	S2	16	−	8	−	−	<1:80
215	−	<10	+	S2	16	−	16	−	−	<1:80
216	−	<10	−	S1	−	−	8	−		
217	−	<10	+	S2	16	−	16	−	−	<1:80
218	−	<10	+	S2	16	−	16	−	−	<1:80
219	−	<10	−	S1	−	−	16	−		
220	−	<10	−	S1	−	−	16	−		
221	−	<10	−	S1	−	−	16	−		
222	−	<10	−	S1	−	+	32	−	−	<1:80
223	−	<10	−	S1	−	−	16	−		
224	−	<10	−	S1	−	−	16	−		
225	−	<10	+	S2	16	+	32	−	−	<1:80
226	−	<10	+	S2	16	−	8	−	−	<1:80
227	−	<10	+	S2	16	+	32	−	−	<1:80
228	−	<10	−	S1	−	−	16	−		
229	−	<10	−	S1	−	+	32	−	−	<1:80
230	−	<10	−	S1	−	+	32	−	−	<1:80
231	−	<10	−	S1	−	−	8	−		
232	−	<10	−	S1	−	−	8	−		
233	−	<10	−	S1	−	−	≤4	−		
234	−	<10	−	S1	−	−	≤4	−		
235	−	<10	−	S1	−	−	≤4	−		
236	−	<10	−	S0	−	−	≤4	−		
237	−	<10	−	S0	−	−	≤4	−		
238	−	<10	−	S0	−	−	≤4	−		
239	−	<10	−	S0	−	−	≤4	−		
240	−	<10	−	S1	−	−	≤4	−		
241	−	<10	+	S3	32	−	16	−		
242	−	<10	+	S3	32	−	16	−		
243	−	<10	+	S2	16	+	48	−		
244	−	<10	+	S2	16	+	48	−		
245	−	<10	+	S2	16	+	32	−		
246	−	<10	+	S3	32	−	16	−		
247	+	10	+	S3	32	−	16	−		
248	−	<10	+	S2	16	+	32	−		
249	−	<10	−	S1	−	−	16	−		
250	−	<10	−	S1	−	+	32	−		
251	+	10	−	S1	−	−	16	−		
252	+	20	+	S2	16	+	48	−		
253	−	<10	+	S2	16	+	32	−		
254	−	<10	−	S1	−	+	32	−		
255	+	10	+	S2	16	+	48	−		
256	−	<10	+	S2	16	+	48	−		
257	−	<10	+	S4	64	−	16	−		
258	−	<10	+	S3	32	−	16	−		
259	−	<10	−	S1	−	+	32	−		
260	+	10	+	S3	32	+	32	−		

+, positive; − negative; S0–S6, scores corresponding to the CombScale of POCT-1.

**Table A5:** Results for the detection of antibodies against CAV, detected by VN and three different POCTs for all dogs (*n* = 260). The reciprocal value of the highest dilution, at which no cytopathic effect was observed, corresponds to the antibody titer.

Sample ID	VN		POCT-1			POCT-2		POCT-3
1	+	80	+	S5	64	+	256	+
2	−	<10	+	S4	32	+	128	−
3	+	40	+	S3	16	+	32	+
4	+	20	+	S4	32	+	64	+
5	+	160	+	S5	64	+	512	−
6	+	160	+	S5	64	+	512	+
7	+	320	+	S4	32	+	64	−
8	+	20	+	S3	16	−	8	−
9	+	160	+	S3	16	+	64	−
10	+	80	+	S4	32	+	256	+
11	+	40	+	S4	32	+	64	+
12	+	40	+	S5	64	+	512	+
13	−	<10	+	S4	32	+	256	−
14	+	160	+	S4	32	+	64	+
15	+	40	+	S4	32	+	64	−
16	+	80	+	S5	64	+	256	+
17	+	320	+	S4	32	+	64	+
18	+	40	+	S4	32	+	128	+
19	+	40	+	S4	32	+	128	+
20	+	40	+	S3	16	+	64	+
21	+	20	+	S4	32	+	128	−
22	+	20	−	S1	−	−	8	+
23	−	<10	+	S3	16	+	16	+
24	+	20	+	S4	32	+	64	+
25	+	80	+	S4	32	+	128	+
26	+	320	+	S3	16	+	64	−
27	+	640	+	S6	≥128	+	512	+
28	+	160	+	S4	32	+	16	−
29	−	<10	invalid			−	8	−
30	−	<10	−	S1	−	−	≤2	−
31	+	80	+	S4	32	+	128	−
32	+	80	+	S2	8	+	16	−
33	+	20	+	S4	32	+	128	−
34	−	<10	+	S2	8	−	≤2	−
35	−	<10	+	S2	8	−	4	−
36	+	160	+	S5	64	+	1024	−
37	+	2560	+	S5	64	+	1024	−
38	+	320	+	S5	64	+	512	+
39	+	10	+	S3	16	+	16	−
40	+	80	+	S3	16	+	256	−
41	+	80	+	S4	32	+	256	+
42	−	<10	+	S3	16	+	16	−
43	−	<10	+	S2	8	+	32	−
44	+	160	+	S4	32	+	512	−
45	+	320	+	S4	32	+	512	−
46	+	20	+	S4	32	+	16	−
47	+	20	+	S3	16	+	32	−
48	+	20	+	S4	32	+	64	−
49	+	160	+	S4	32	+	256	−
50	+	10	+	S3	16	+	128	−
51	+	1280	+	S5	64	+	1024	−
52	−	<10	+	S4	32	+	128	−
53	+	20	+	S3	16	+	32	−
54	+	20	+	S4	32	+	64	−
55	−	<10	+	S3	16	+	16	−
56	+	80	+	S4	32	+	16	−
57	−	<10	+	S4	32	−	4	−
58	−	<10	+	S2	8	−	4	−
59	−	<10	+	S3	16	−	4	−
60	+	80	+	S4	32	+	64	+
61	+	160	+	S4	32	+	256	−
62	−	<10	−	S1	−	−	≤2	−
63	+	320	+	S3	16	+	128	−
64	+	40	+	S4	32	+	128	−
65	+	10	+	S3	16	+	64	−
66	+	160	+	S4	32	+	64	−
67	+	40	+	S3	16	+	128	−
68	+	80	+	S4	32	+	128	+
69	+	80	+	S4	32	+	256	−
70	+	20	+	S3	16	+	16	−
71	−	<10	+	S2	8	−	8	−
72	+	10	+	S3	16	+	32	−
73	+	320	+	S5	64	+	256	−
74	+	160	+	S5	64	+	512	+
75	+	40	+	S4	32	+	64	+
76	+	10	+	S3	16	−	8	+
77	+	40	+	S5	64	+	256	−
78	−	<10	+	S2	8	−	≤2	−
79	+	20	+	S3	16	+	32	−
80	+	80	+	S5	64	+	256	−
81	+	40	+	S4	32	+	64	−
82	+	160	+	S4	32	+	16	−
83	+	640	+	S6	≥128	+	1024	−
84	+	160	+	S5	64	+	512	+
85	+	160	+	S5	64	+	512	−
86	+	40	+	S6	≥128	+	1024	−
87	+	80	+	S4	32	+	64	−
88	+	160	+	S5	64	+	256	−
89	−	<10	+	S2	8	+	16	−
90	+	20	+	S4	32	+	16	−
91	−	<10	+	S3	16	+	32	+
92	+	160	+	S4	32	+	256	−
93	+	10	+	S4	32	+	128	−
94	−	<10	+	S2	8	−	4	−
95	+	20	+	S4	32	+	64	+
96	+	160	+	S4	32	+	128	−
97	+	80	+	S4	32	+	128	−
98	+	160	+	S5	64	+	128	−
99	+	40	+	S4	32	+	32	−
100	+	20	+	S2	8	−	8	+
101	−	<10	+	S3	16	−	8	+
102	+	20	+	S4	32	+	16	−
103	+	40	+	S4	32	+	64	+
104	−	<10	+	S3	16	+	64	+
105	+	2560	+	S4	32	+	512	−
106	+	80	+	S5	64	+	256	−
107	+	10	+	S2	8	−	8	−
108	+	20	+	S2	8	−	4	+
109	+	20	+	S5	64	+	128	+
110	+	640	+	S6	≥128	+	1024	−
111	+	40	+	S3	16	+	32	−
112	+	40	+	S3	16	+	32	+
113	+	40	+	S4	32	+	16	+
114	+	640	+	S6	≥128	+	1024	+
115	+	80	+	S4	32	+	64	−
116	+	10	+	S5	64	+	128	−
117	+	80	+	S4	32	+	128	−
118	+	40	+	S4	32	+	64	−
119	+	80	+	S5	64	+	128	−
120	+	2560	+	S5	64	+	512	−
121	+	160	+	S6	≥128	+	512	+
122	−	<10	+	S4	32	+	32	−
123	+	40	+	S4	32	+	32	−
124	−	<10	+	S6	≥128	+	1024	−
125	+	640	+	S6	≥128	+	1024	+
126	+	40	+	S6	≥128	+	256	+
127	+	320	+	S5	64	+	128	−
128	+	10	+	S3	16	+	16	−
129	+	10	+	S3	16	+	32	−
130	+	160	+	S4	32	+	64	−
131	+	160	+	S5	64	+	256	−
132	+	20	+	S4	32	+	32	−
133	+	320	+	S5	64	+	256	+
134	+	80	+	S5	64	+	64	+
135	−	<10	+	S4	32	+	128	−
136	+	20	+	S3	16	−	4	−
137	+	80	+	S4	32	−	4	−
138	+	80	+	S5	64	+	64	−
139	+	1280	+	S6	≥128	+	256	−
140	−	<10	+	S3	16	+	16	−
141	−	<10	+	S3	16	−	4	+
142	+	80	+	S4	32	+	64	−
143	+	20	+	S2	8	−	≤2	+
144	+	160	+	S5	64	+	256	+
145	−	<10	+	S2	8	−	4	−
146	+	160	+	S5	64	+	512	−
147	+	320	+	S4	32	+	256	+
148	+	10	+	S2	8	−	8	+
149	−	<10	+	S3	16	+	16	−
150	+	640	+	S5	64	+	512	+
151	+	20	+	S3	16	+	32	+
152	+	20	+	S4	32	+	64	−
153	+	10	+	S3	16	+	16	−
154	+	20	+	S3	16	+	16	−
155	+	80	+	S5	64	+	256	−
156	+	80	+	S5	64	+	256	+
157	−	<10	+	S3	16	−	4	−
158	+	80	+	S5	64	+	1024	−
159	+	40	+	S5	64	+	512	−
160	−	<10	+	S4	32	+	128	−
161	+	80	+	S6	≥128	+	512	−
162	−	<10	−	S1	−	−	≤2	−
163	+	320	+	S6	≥128	+	512	−
164	+	40	+	S4	32	+	16	−
165	−	<10	+	S2	8	−	≤2	−
166	+	320	+	S4	32	+	256	+
167	+	10	+	S4	32	+	32	−
168	+	160	+	S5	64	+	256	−
169	+	40	+	S5	64	+	128	−
170	+	40	+	S4	32	+	64	−
171	+	160	+	S3	16	−	8	+
172	−	<10	+	S2	8	−	≤2	−
173	+	20	+	S4	32	+	16	+
174	+	160	+	S4	32	+	64	−
175	+	1280	+	S5	64	+	1024	+
176	+	10	+	S5	64	+	128	−
177	+	160	+	S6	≥128	+	512	−
178	+	20	+	S3	16	+	16	−
179	+	40	+	S5	64	+	128	+
180	+	320	+	S5	64	+	512	+
181	+	320	+	S5	64	+	512	+
182	+	20	+	S5	64	+	512	−
183	−	<10	+	S2	8	−	≤2	+
184	−	<10	+	S3	16	−	8	−
185	+	160	+	S4	32	−	4	−
186	+	320	+	S4	32	+	256	−
187	+	1280	+	S5	64	+	256	−
188	+	40	+	S4	32	+	128	−
189	+	80	+	S4	32	+	128	−
190	+	160	+	S5	64	+	512	−
191	+	10	+	S3	16	−	8	+
192	+	640	+	S4	32	+	512	−
193	+	80	+	S4	32	+	512	−
194	+	640	+	S6	≥128	+	1024	−
195	+	40	+	S3	16	−	8	−
196	+	10	+	S3	16	+	32	−
197	+	20	+	S4	32	+	128	+
198	+	20	+	S2	8	−	4	−
199	+	20	+	S3	16	+	32	−
200	−	<10	+	S2	8	−	≤2	−
201	−	<10	−	S0	−	−	≤2	−
202	−	<10	−	S0	−	−	4	+
203	−	<10	−	S0	−	−	≤2	−
204	−	<10	−	S0	−	−	≤2	−
205	−	<10	−	S0	−	−	≤2	−
206	−	<10	−	S0	−	−	4	−
207	−	<10	−	S0	−	−	≤2	−
208	−	<10	−	S0	−	−	≤2	−
209	−	<10	−	S0	−	−	≤2	−
210	−	<10	−	S0	−	−	≤2	−
211	−	<10	−	S0	−	−	≤2	−
212	−	<10	−	S0	−	−	≤2	+
213	−	<10	−	S0	−	−	4	−
214	−	<10	−	S0	−	−	≤2	−
215	−	<10	+	S3	16	−	8	−
216	−	<10	−	S0	−	−	≤2	−
217	−	<10	−	S0	−	−	≤2	−
218	−	<10	−	S0	−	−	≤2	−
219	−	<10	−	S0	−	−	≤2	−
220	−	<10	−	S0	−	−	≤2	+
221	−	<10	−	S0	−	−	≤2	−
222	−	<10	−	S0	−	−	≤2	−
223	−	<10	−	S0	−	−	≤2	−
224	−	<10	−	S0	−	−	≤2	−
225	−	<10	−	S1	−	−	≤2	−
226	−	<10	−	S0	−	−	≤2	−
227	−	<10	−	S0	−	−	≤2	−
228	−	<10	−	S0	−	−	≤2	+
229	−	<10	−	S0	−	−	8	−
230	−	<10	−	S0	−	−	4	+
231	−	<10	−	S0	−	−	≤2	−
232	−	<10	−	S0	−	−	≤2	−
233	−	<10	−	S0	−	−	≤2	−
234	−	<10	−	S0	−	−	≤2	−
235	−	<10	−	S0	−	−	≤2	−
236	−	<10	−	S0	−	−	≤2	−
237	−	<10	−	S0	−	−	≤2	−
238	−	<10	−	S0	−	−	≤2	−
239	−	<10	−	S0	−	−	≤2	−
240	−	<10	−	S0	−	−	≤2	−
241	+	10	+	S4	32	+	32	−
242	−	<10	+	S4	32	+	64	−
243	+	40	+	S4	32	+	64	−
244	+	40	+	S4	32	+	128	−
245	−	<10	−	S1	−	−	≤2	−
246	+	10	+	S4	32	+	32	−
247	+	10	−	S1	−	−	≤2	−
248	−	<10	+	S3	16	−	8	−
249	+	20	+	S4	32	−	8	−
250	+	20	+	S3	16	−	4	−
251	+	40	+	S3	16	+	16	−
252	+	80	+	S4	32	+	64	−
253	−	<10	+	S2	8	−	8	−
254	−	<10	+	S2	8	−	4	−
255	+	80	+	S4	32	+	64	−
256	+	10	+	S3	16	−	8	−
257	+	10	+	S4	32	+	64	−
258	+	10	+	S4	32	+	64	−
259	−	<10	+	S2	8	−	4	−
260	−	<10	+	S2	8	−	≤2	−

+, positive; − negative; S0–S6, scores corresponding to the CombScale of POCT-1.
